# The development process of ‘fit-for-purpose’ imaging biomarkers to characterize the tumor microenvironment

**DOI:** 10.3389/fmed.2024.1347267

**Published:** 2024-05-16

**Authors:** Jakoba J. Eertink, Idris Bahce, John C. Waterton, Marc C. Huisman, Ronald Boellaard, Andreas Wunder, Andrea Thiele, Catharina W. Menke-van der Houven van Oordt

**Affiliations:** ^1^Department of Medical Oncology, Amsterdam UMC Location Vrije Universiteit Amsterdam, Amsterdam, Netherlands; ^2^Imaging and Biomarkers, Cancer Center Amsterdam, Amsterdam, Netherlands; ^3^Department of Pulmonary Medicine, Amsterdam UMC Location Vrije Universiteit Amsterdam, Amsterdam, Netherlands; ^4^Centre for Imaging Sciences, University of Manchester, Manchester, United Kingdom; ^5^Department of Radiology and Nuclear Medicine, Amsterdam UMC Location Vrije Universiteit Amsterdam, Amsterdam, Netherlands; ^6^Department of Translational Medicine and Clinical Pharmacology, Boehringer Ingelheim Pharma GmbH & Co. KG, Biberach and der Riss, Germany

**Keywords:** imaging biomarker, PET imaging, MR imaging, context of use, tumor microenvironment, validation

## Abstract

Immune-based treatment approaches are successfully used for the treatment of patients with cancer. While such therapies can be highly effective, many patients fail to benefit. To provide optimal therapy choices and to predict treatment responses, reliable biomarkers for the assessment of immune features in patients with cancer are of significant importance. Biomarkers (BM) that enable a comprehensive and repeatable assessment of the tumor microenvironment (TME), the lymphoid system, and the dynamics induced by drug treatment can fill this gap. Medical imaging, notably positron emission tomography (PET) and magnetic resonance imaging (MRI), providing whole-body imaging BMs, might deliver such BMs. However, those imaging BMs must be well characterized as being ‘fit for purpose’ for the intended use. This review provides an overview of the key steps involved in the development of ‘fit-for-purpose’ imaging BMs applicable in drug development, with a specific focus on pharmacodynamic biomarkers for assessing the TME and its modulation by immunotherapy. The importance of the qualification of imaging BMs according to their context of use (COU) as defined by the *Food and Drug Administration* (*FDA*) *and National Institutes of Health Biomarkers, EndpointS, and other Tools* (*BEST*) glossary is highlighted. We elaborate on how an imaging BM qualification for a specific COU can be achieved.

## Introduction

Immune-based therapies have revolutionized cancer treatment. However, tumor responses to such therapies are dependent on specific features of the tumor microenvironment (TME). The TME is a complex environment that is constantly interacting with the tumor cells. It consists of blood vessels, stromal components, and different cell types such as immune cell subsets (1, 2). Successful development of immune-based treatments requires an understanding of the tumor biology, including its TME, as well as of the contribution of the lymphoid system (2). The modulation of the TME features and its interplay with the components of the immune system as induced by specific treatments are crucial for the efficacy of the treatment. Biomarkers (BMs) quantifying such changes can be used to assess pharmacodynamic aspects such as the mode of action of the drug, to predict immunotherapy efficacy, to provide information on immunotherapy resistance, and thus for developing novel immunotherapy strategies.

To evaluate the composition of the TME, tumor biopsies are the current standard. However, such biopsies are invasive and provide only small tissue samples for a very limited number of lesions, usually just one. As the TME has a heterogeneous composition within and between tumor lesions and, in addition, no information is provided for the lymphoid tissues, important sites involved in the immune response, the interpretability of results from biopsies is rather limited.

Imaging BMs may overcome these limitations, as imaging modalities such as positron emission tomography (PET) or magnetic resonance imaging (MRI) can offer a minimally invasive and comprehensive solution by providing whole-body data. PET is an imaging technique that uses radiolabeled compounds (radiotracers) to visualize specific physiological and pathophysiological processes at a molecular level, whereas MRI is another medical imaging technique that generates detailed images of anatomical structures and physiological processes, such as perfusion. Therefore, all detectable tumor lesions, their associated TMEs, and also lymphoid tissues can be assessed. As imaging BMs can be applied repeatedly, valuable insights on the development of tumor lesion and their TMEs and treatment effects can be gained over time. This enables optimization of treatments as well as patient selection and can address resistance mechanisms. Therefore, imaging BMs can strongly support advancements in cancer immunotherapy. However, imaging BMs need to be characterized thoroughly before robust inferences about the TMEs and lymphoid tissues can be drawn.

This review focuses on the development cascade of ‘fit-for-purpose’ quantitative imaging BMs, i.e., biomarkers with a level of validation sufficient to support its exploratory application in a defined context of use (COU). The COU is a framework provided through the FDA “Biomarkers, Endpoints, and Other Tools (BEST)” initiative and defines the BM category and the BM’s intended use for application in drug development (3). Imaging BMs delivering information on (patho) physiological states and processes such as tissue composition, blood flow and volume, water content, distribution, (changes of) expression, and availability, respectively, of specific TME-related targets will be discussed. Moreover, we address the need to undertake biological, clinical, and technical validation steps, including the acquisition of data required to assure the generation of reliable and repeatable imaging BMs ‘fit-for-purpose’ for the specific COU. Furthermore, the need for standardization and harmonization to be able to use ‘fit-for-purpose’ quantitative imaging BMs in multicenter trials will be addressed. While the COU criteria help qualifying a BM for regulatory use, such criteria can also support the validation for exploratory BMs aiming to be used for decision-making, for instance, in early clinical drug development. Such exploratory BMs need to have a minimum level of validation sufficient to confirm their reliability and accuracy for the intended purpose, i.e., COU, ensuring they are ‘fit-for-purpose’. Of note, requirements for a regulatory approval of a BM will not be discussed. Such BMs undergo a multi-step development process following defined tightly regulated processes and are not in the scope of this review.

The review extends the “imaging biomarker roadmap for cancer studies” published in 2017 by O’Connor et al. (4) and refers to recommendations published by the *Society of Nuclear Medicine and Molecular Imaging* (SNMMI), *European Association of Nuclear Medicine* (EANM), *European Society of Radiology* (ESR), and the *Quantitative Imaging Biomarker Alliance of the Radiological Society of North America* (QIBA) (5–8). It focuses on imaging BMs to assess pharmacodynamic processes in particular and the related specific aspects to successfully perform the validation steps needed to cross the “first translational gap” described by O’Connor et al. (4). The review should encourage a joint discussion aiming at an alignment on the assessments and data sets needed to conclude whether an imaging BM can be regarded as ‘fit-for-purpose’ for an application as an exploratory pharmacodynamic (PD) imaging BM in early clinical trials.

## The tumor microenvironment (TME)

### Key targets for predicting immunotherapy efficacy and resistance to immunotherapy

Proliferating tumor cells profoundly influence their microenvironment by initiating a number of pathophysiological processes, thereby generating a protective milieu, the TME, which further promotes tumor development. The resulting complex TME consisting of components such as tumor neovasculature, immune cells, fibroblasts, signaling molecules, and extracellular matrix, influences the immune signature of the lesion and is critical for tumor survival and growth. Moreover, the TME composition can vary within and between the tumor lesions of a single patient (1).

The most relevant cell populations in the TME influencing a response to immune-based therapies are T-cell subtypes, i.e., CD8^+^ T cells, cytotoxic cells (important for an effective immune response), CD4^+^ T helper cells and T regulatory cells (both can inhibit immune responses). Furthermore, myeloid cells including macrophages, cancer-associated fibroblasts, and cells driving cancer neoangiogenesis are present. In addition, pathophysiological factors such as hypoxia, acidosis, and perfusion affect the composition of the TME and its ability to respond to immune modulatory treatments. The current understanding is that there are three major phenotypes of the TME. The first comprises of a high degree of T cells, which can be activated and expanded (hot immune/ inflamed tumors). The second milieu is either restrictive with sparse infiltration of immune cells or has an overwhelming presence of immune suppressive cells. It may also exclude immune cells from the tumor core by a physical barrier, such as the presence of hypoxia and aberrant tumor vasculature (altered-immunosuppressed immune tumors/T-cell exclusion). The third milieu prevents the presence of any immune cells (cold immune tumors/T-cell desert) (9, 10). The plasticity of the TME including the processes leading to modulation toward different phenotypes until now is largely unknown. However, understanding these processes involved will be essential for optimal drug development.

**Figure 1:**
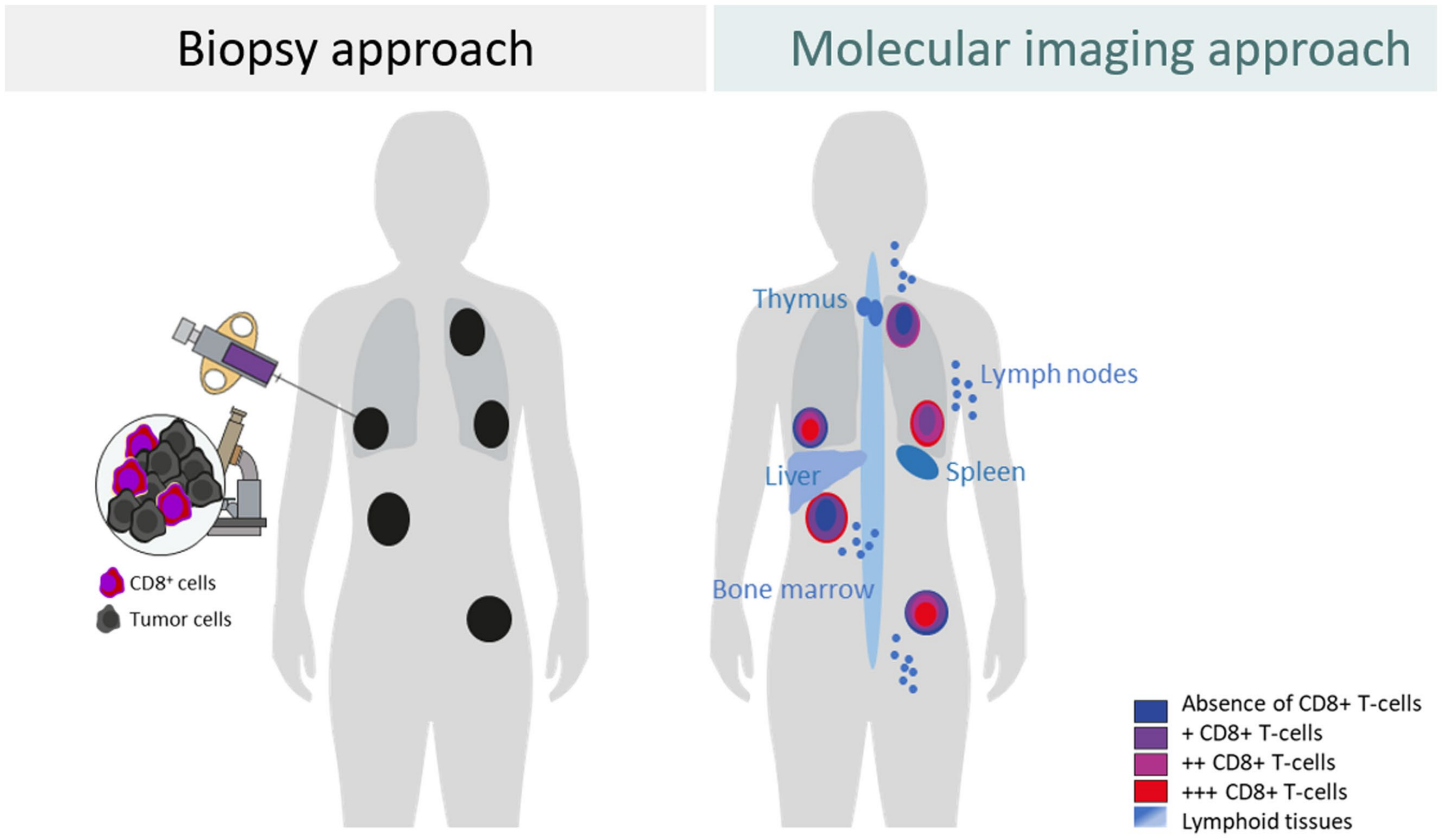
Differences between a biopsy approach and a molecular imaging approach to assess the tumor and tumor microenvironment exemplified for CD8^+^ T cells.

Although tumor biopsy-derived BMs provide information on the TME and support therapeutic choices for patients, they have significant shortcomings. It is neither possible to assess the features of each part of a tumor lesion nor of every tumor lesion and its related TME in a body. Additionally, repeated biopsies are difficult to achieve. Moreover, to understand the contribution of the systemic immune response in the context of cancer immunotherapy, assessment of the lymphoid tissues such as the spleen, lymph nodes, and bone marrow can be important. As the biopsy-derived biomarkers have not been shown to optimally assess drug-induced changes in the TMEs (11, 12), whole-body imaging BMs may fill this gap and may help to discover not yet fully understood causes of why treatment fails to eradicate tumor cells in some patients and some tumor lesions, while others respond quickly and completely (Figure 1).

### Imaging BMs to assess the TME: current gaps and possible solution

In contrast to biopsies, computed tomography (CT), MRI, or PET imaging can detect pathological, pathophysiological, and molecular changes, repeatedly, with high spatial resolution, providing integral whole-body information. All three imaging modalities can deliver quantitative data for tumor lesions approximately 1 cm in size. Current clinical applications include structural CT for the determination of tumor size, contrast-enhanced MRI for the assessment of vascular permeability, or [^18^F]fludeoxyglucose (FDG) PET for the detection of tumor glycolysis.

**Figure 2:**
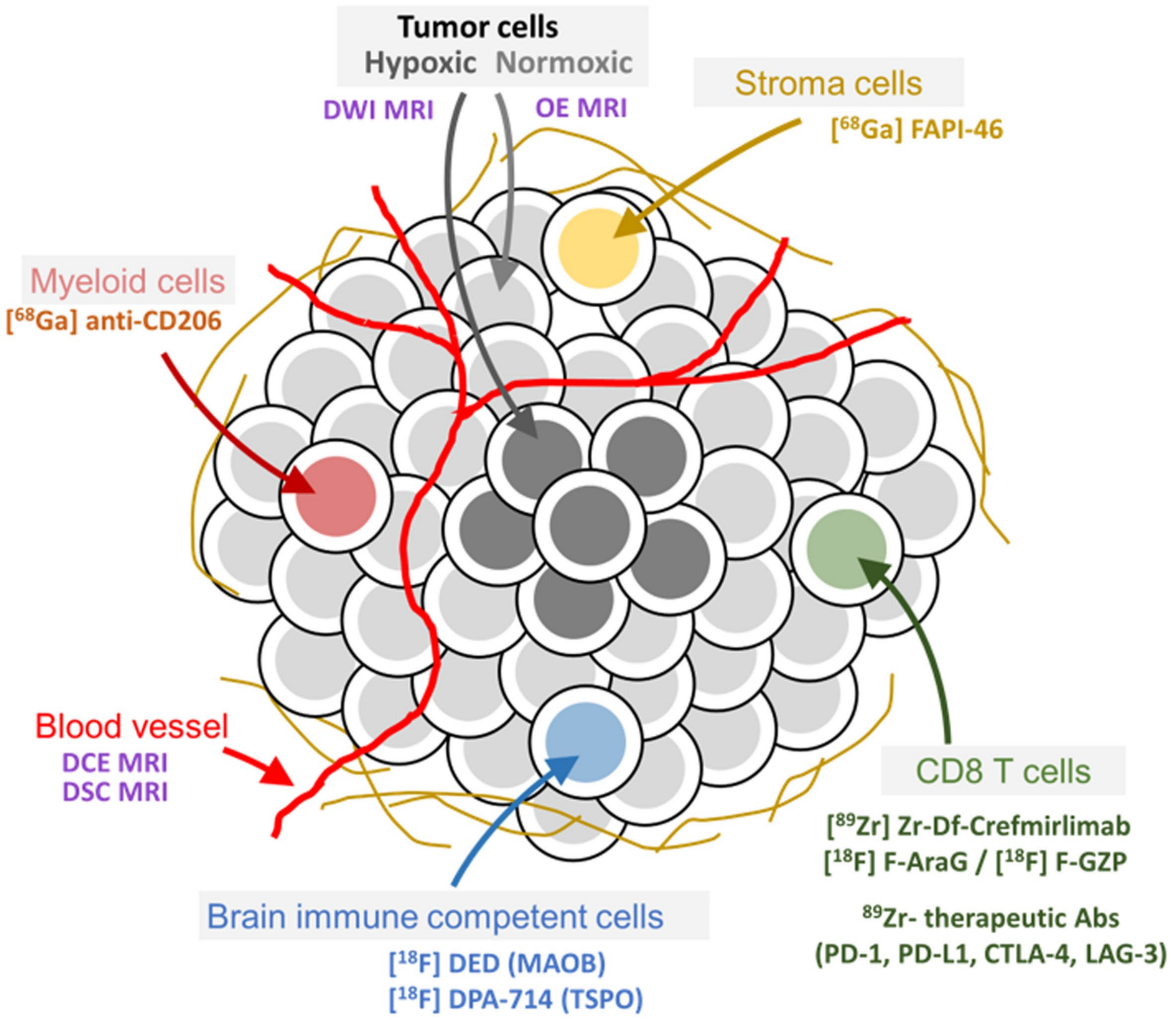
Tumor and tumor microenvironment composition and related imaging methods to assess pathophysiological and molecular features. Examples of well-characterized PET ligands and imaging methods for specific targets and physiological processes, respectively, in the tumor microenvironment of peripheral as well as brain tumors, are depicted in the figure.

**Figure 3:**
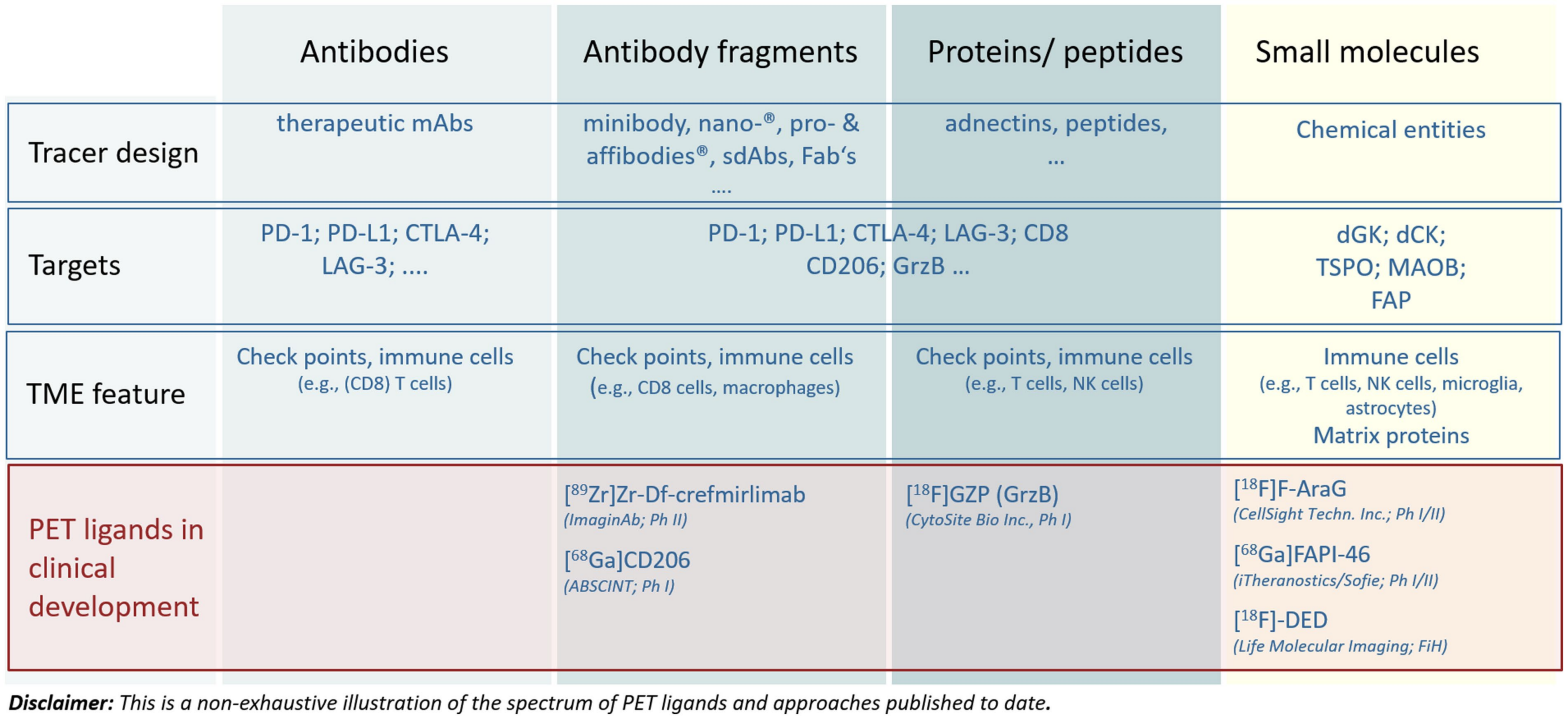
Main approaches to address targets in the tumor microenvironment by molecular imaging. The figure illustrates the diversity of compound entities applied for different targets as well as the most advanced PET tracers undergoing clinical development. For more details see the articles of Swenck et al. (13) and Slebe et al. (14).

CT and MRI are already highly integrated in clinical drug development and daily practice. For morphological features, CT and MRI provide well-characterized imaging BMs such as tumor size (RECIST v1.1; RANO) as well as features such as shape, density, cellularity, and perfusion. However, RECIST v1.1-based tumor size assessment can be misleading in early drug development if the treatment causes stasis or if a transient flare is mistaken for progressive disease. In addition, morphological changes occur late and do not provide insights into changes occurring in tumor tissues that might lead to morphological changes. In order to steer drug development, imaging BMs are needed that can be measured early after treatment initiation for understanding pharmacodynamic changes within the TME induced by the drug. For instance, specific PET ligands, i.e., radiolabeled molecules, mainly biologics, with the potential to decipher specific molecular characteristics of the TME are currently developed (Figures 2, 3). Those approaches have great potential to generate highly informative quantitative imaging BMs for every single evaluable tumor lesion, the related TME(s), and the lymphoid compartments. Thus, imaging BMs related to such physiological and molecular changes are particularly helpful as pharmacodynamic BMs in early drug development. In later drug development, those imaging BMs could help to get a better understanding of pathophysiological/molecular resistance mechanisms such as PD-L1 expression, CD8^+^ cell exclusion, or functional exhaustion of immune cells impeding anti-tumor responses (15, 16).

With the development of immune-oncological treatments, the importance of assessing changes triggered in the TMEs and those occurring in the immune compartments of the body increases. The integration of both into informative BM readouts will be important for a comprehensive characterization of the patient’s immune state.

Recently, [^89^Zr]crefmirlimab berdoxam PET-MRI has been used to provide data integrating CD8^+^ cell changes in both tumor lesions and lymphoid tissues, with changes in tumor cellularity as derived from diffusion-weighted MRI. These data impressively illustrate the potential to build unique and powerful sets of biomarkers for a comprehensive assessment of the mode of action of investigational drugs during clinical development and beyond (17, 18).

Importantly, such imaging BMs must be appropriately characterized. To better understand pharmacodynamic changes such as those induced by the mode of action of the drugs and provide response patterns for decision-making in drug development, reliable and reproducible ‘fit-for-purpose’ imaging BMs are needed.

### The use of imaging biomarkers to assess the TME

An imaging BM is a measurement that is derived from medical images gathered by dedicated medical imaging devices combined with an application of specific acquisition, image reconstruction, and processing algorithms. In case of PET, a radiolabeled ligand, also called tracer, is needed, while CT and MRI often require a contrast agent. Radiolabeled ligands and contrast agents are regulated as drugs and need to be produced under regulated conditions. In addition, clinical imaging BMs rely on certified hardware and software such as scanners, algorithms, and imaging acquisition sequences (4, 6, 19–21). How to use the imaging modalities and which PD imaging BMs are intended to be derived, depend on the pathophysiology of the disease, the specific treatments, and their mode of action, as well as on the COU (22).

Imaging BMs such as the transfer coefficient K^trans^ for perfusion, the apparent diffusion coefficient for tumor cellularity measured by MRI, or measures of tumor glucose consumption using [^18^F]FDG PET can be relevant to the TME. These imaging BMs, when properly applied are already ‘fit-for-purpose’ for application in early-phase clinical trials, but are not particularly specific for immunotherapy (23, 24).

Many PET ligands ranging in size from ^11^C- or ^18^F-labeled small molecules to ^89^Zr-labeled therapeutic antibodies targeting molecular features of the TME have been described (Figures 2, 3) (13, 14, 25–27). They provide impressive insights into TME’s enabling assessment of intralesional heterogeneity as well as of the tumor lesion heterogeneity within and between patients and its modulation caused by a pharmacological intervention (17, 28–30). However, only a few tracers are currently undergoing rigorous clinical qualification and validation processes aiming at generating fully validated quantitative imaging BMs for diagnostic applications (5, 20, 31). Figure 3 presents a non-exhaustive illustration of radiolabeled compounds targeting TMEs published to date. The reader is referred to recent reviews for more extensive lists of tracers being developed (13, 14).

**Figure 4:**
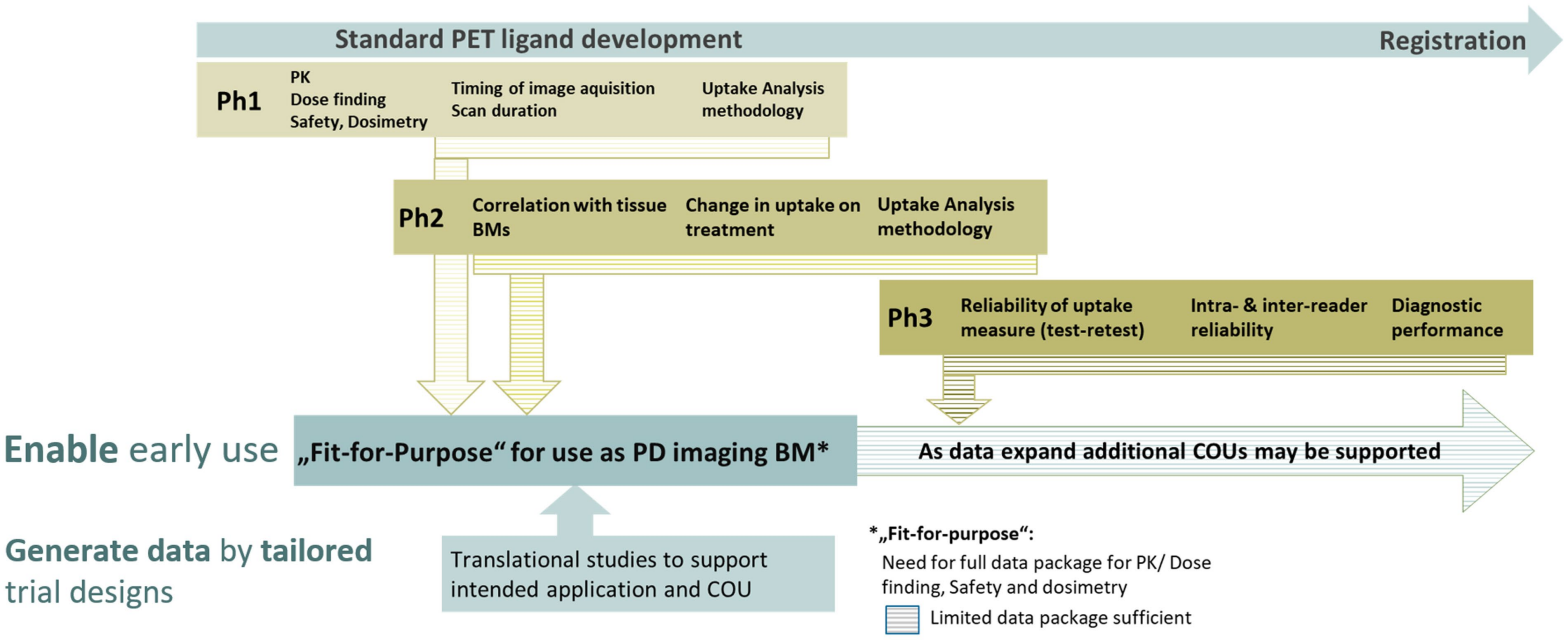
Schematic view on the main assessments occurring in the different phases of a PET ligand development. The validation of imaging biomarkers is based on data generated at different time points through standard PET development phases. This can be supported by dedicated translational studies enabling the timely application of novel PET ligands and the derived imaging biomarkers for clinical development decisions even before a PET ligand has been fully developed.

As PET ligands for clinical use are regulated as diagnostic drugs, they must undergo a defined clinical development process. This process comprises different well-known clinical phases and can take 8 to 10 years before approval through a new drug application (NDA) or marketing authorization application (MAA). It is a multi-step and iterative process addressing parameters such as sensitivity, specificity, repeatability, accuracy, and diagnostic performance of the derived imaging BMs (4–6, 19, 21). For illustration please see Figure 4.

In early drug development, a PET ligand from which a PD imaging BM is derived, however, does not require regulatory approval. For example, in neuroscience, PET ligands such as [^11^C]raclopride or [^11^C]flumazenil have been used successfully for decades in pharmacodynamic studies without NDA/MAA approval (32, 33). Furthermore, amino acid PET ligands, such as [^18^F]fluoroethyltyrosine, are increasingly used in patients with glioma (34).

O’Connor *et al* published a consensus statement on the minimum requirements for an imaging BM to be applied in early drug development (4). To accelerate a successful clinical translation, several steps may occur in parallel including correlation of the imaging BM with the tissue-derived BM, measurement consistency, and defining the imaging BM’s specificity, sensitivity, and repeatability. Such translational steps are required for biospecimen-based biomarkers as well (35). However, the translation and respective steps for imaging BMs require image data generated in patients in well-designed clinical trials, making it more time-consuming, complex, and necessarily iterative.

### The context of use and fit for purpose criteria

**Figure 5:**
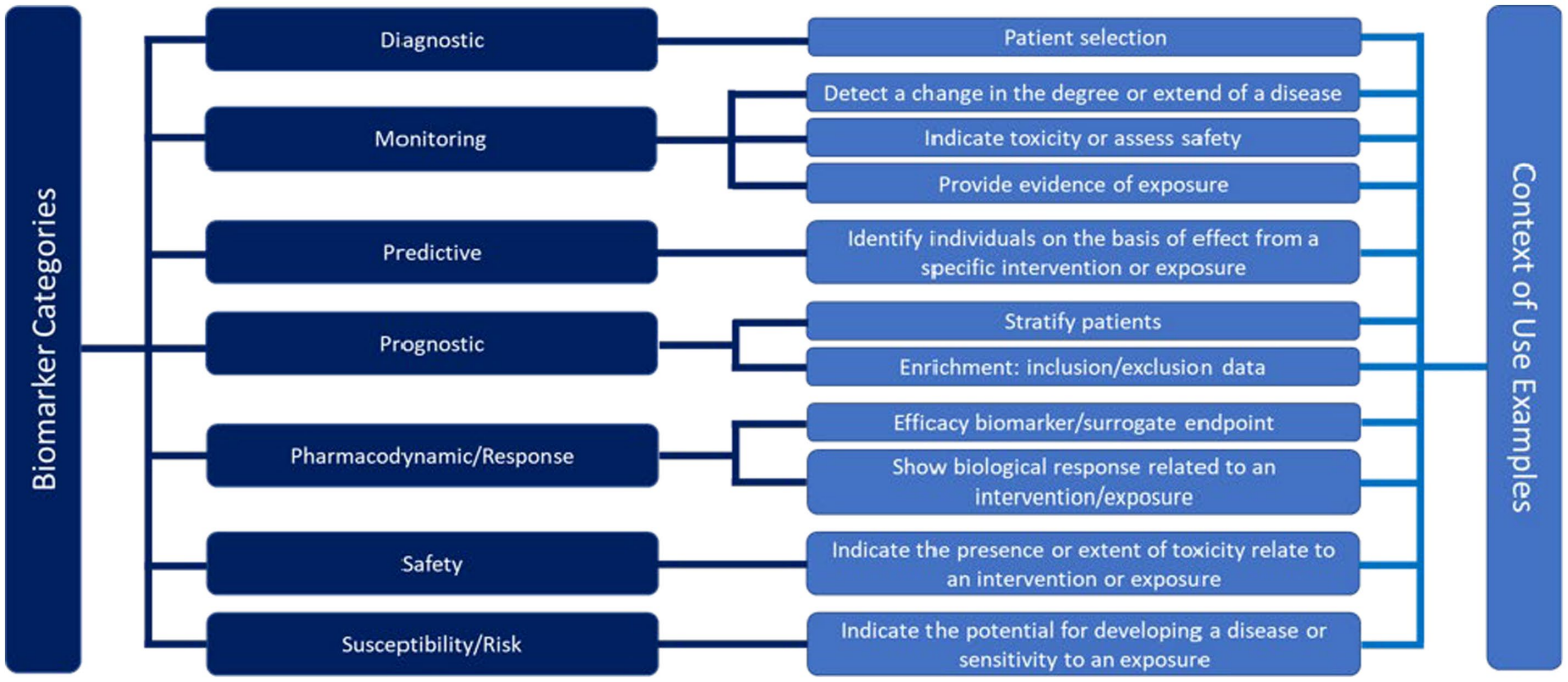
Framework for the definition of the context of use for different biomarker categories in a drug development application according to the Food and Drug Administration (FDA) (3).

The use of biomarker in drug development is concisely described. The criteria from the FDA provide the framework for the definition of the COU for every biomarker in a drug development application (Figure 5). The FDA has established a specialized biomarker qualification process based on the COU criteria including “the BM category and the BM’s intended use in drug development” (3) (Biomarker Qualification Program | FDA). In the BEST Resource document (3), the following statement is made: “*The biomarker description is a succinct but comprehensive summary that is intended to correctly identify the biomarker, its biologic plausibility* (i.e.*, relevance to the disease or condition*), *and its measurement method. While not exhaustive, these key features of a biomarker’s description convey important information to assess information from multiple sources* (e.g.*, evaluating results and conclusions from data published by different laboratories*).”

While the COU criteria help to qualify a BM for regulatory use, such criteria can also support the COU of exploratory BMs used for decision-making in drug development. Of note, however, exploratory BMs need a minimum level of characterization regarding their reliability and accuracy for the intended purpose, ensuring they are ‘fit-for-purpose’. During such a process, biological, clinical, and technical performances are assessed. **Biological validation** assures that the imaging BM truly reflects the underlying *in vivo* biology (specificity) and that the measurement method is sensitive enough to detect relevant changes in line with the underlying change of the relevant target in the tissue. **Clinical validation** includes the safe use of the imaging asset in a clinical situation and ensures that the imaging BM can reliably measure the pharmacodynamic change of interest. **Technical validation** concerns kinetics of PET ligand (this also applies to contrast media for CT and MRI) and its comparability among subjects, both, before and during/after the intervention. In addition, the accuracy of the uptake metrics quantifying the underlying biological features and their changes, and methods to normalize the data, assess reproducibility and repeatability are included. Validations of whether simplified semi-quantitative uptake metrics such as standardized uptake values (SUVs) can be utilized may be performed as well (36). Technical validation aims at the reliable performance of procedures and assessments that can be applied in every trial center (37) Of note, early-phase oncology trials with a PD imaging BM can be performed single-center, requiring good same-scanner repeatability, or multicenter requiring good between-scanner reproducibility, scanner harmonization, protocol harmonization, and ideally centralized data analysis using reliable uptake metrics (more details are provided below). Complete technical validation is achieved, when an imaging BM measurement can be performed in a reproducible manner in any required geographical location, providing comparable data. This, however, does not address underlying biology or its relation to clinical outcomes.

### Translating TME features into imaging biomarkers for a COU

**Figure 6:**
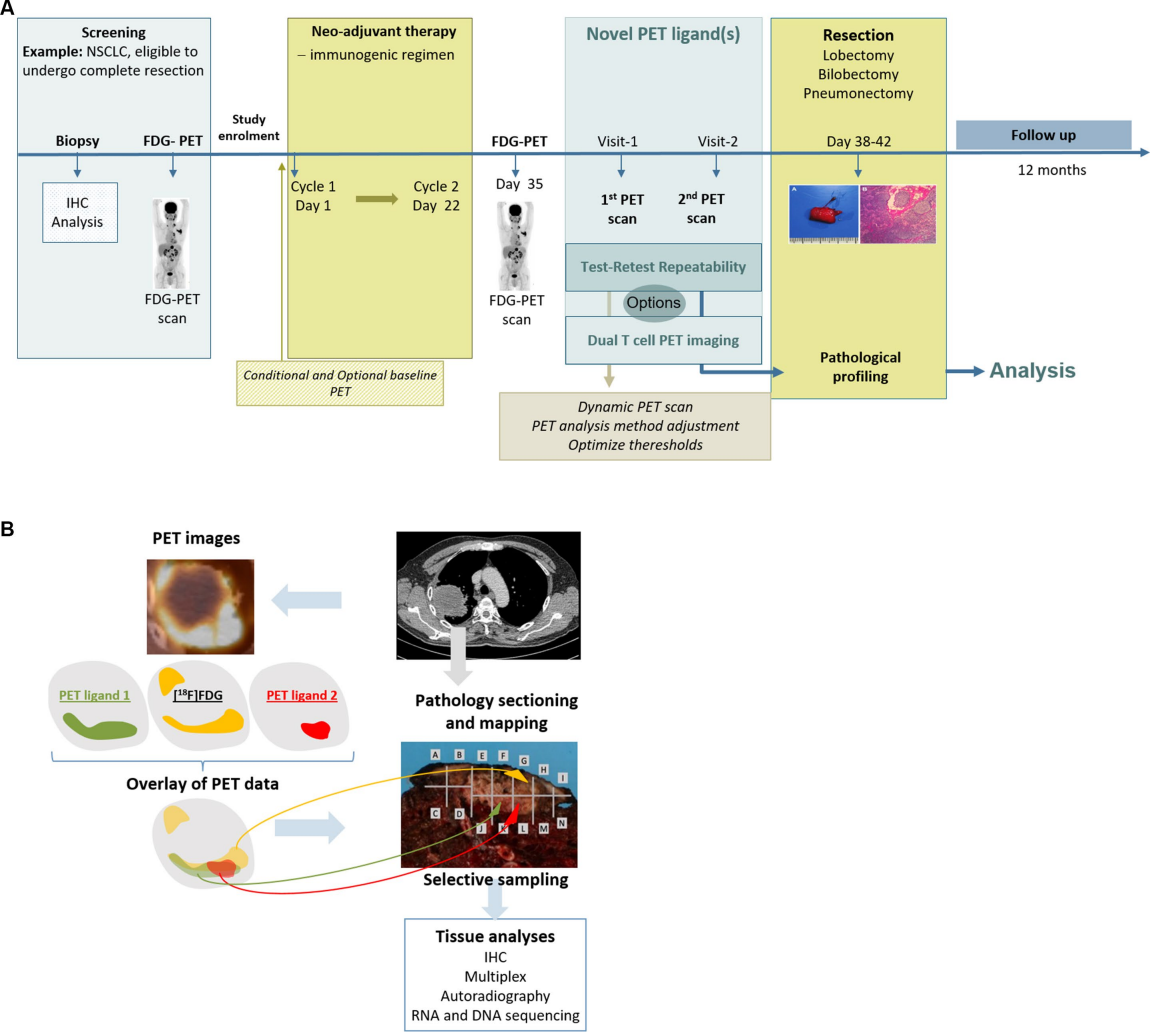
**(A)** Example of a comprehensive and tailored clinical trial design that can fill the gap(s) for an intended COU. **(B)** Example of a comprehensive analysis design for an intended COU by correlating imaging data and pathology data.

This paragraph will focus on the requirements for PET ligands and derived BMs. Requirements for radiological methods have been detailed in publications provided by, e.g., the European Society of Radiology (ESR) and QIBA (5, 6, 21, 38, 39). The biological usefulness of a PET ligand for the intended imaging BM is among others, determined by its affinity and selectivity for the target, specificity of its uptake target availability internalization, and/or recycling as the drivers of its uptake. In addition, the uptake is influenced by its pharmacokinetic (PK) properties, such as distribution, clearance, and tracer metabolism. These features informing on the **biological performance** can be assessed in animal studies. The use of mice bearing, e.g., tumor xenografts with different levels of target expression, allows us to measure uptake specificity and sensitivity. In addition, *in vivo* blocking studies in rodents or non-human primates will provide data with respect to the correlation of the tracer uptake with the underlying biology, i.e., target regulation and availability (40). Furthermore, studies using radiolabeled drugs targeting TME features in patients have the potential to deliver additional quantitative information on target expression, target behavior, and extent of target blockade *in vivo* that *in vitro* or animal data cannot provide (41–43). To assess the accuracy of the tracer uptake, tumor tissue samples can be collected in conjunction with the imaging procedure during the course of a clinical study. However, as elaborated earlier, biopsies are small tumor samples, neglecting tumor lesion heterogeneity and lacking information on target availability and distribution throughout the tumor. This impacts the comparison with imaging features or PET ligand uptake measures typically derived from whole tumor volumes. A path to overcome this is the implementation of medical imaging assessments into studies in patients treated with a neoadjuvant regimen followed by tumor resection. The imaging data can be used to guide sampling from the resected tissue for assessment of the expression of the target of interest within heterogeneous areas. This, in turn, leads to a more comprehensive determination of target heterogeneity, enabling direct assessment of the accuracy of the observed imaging data (Figure 6). In this way, a thorough biological validation with respect to the sensitivity, specificity, and accuracy of the uptake measures can be achieved.

During **clinical validation**, the safety and tolerability of a tracer as well as the radiation burden the tracer will deliver to a patient must be determined. Furthermore, the accuracy and precision, i.e., the degree of consistency or repeatability, of the imaging BM is assessed. The optimal timing of the image acquisition post tracer injection and the necessary scan duration to achieve optimal uptake levels will be informed by the PK profile of the PET ligand. For biologicals, such as antibodies or minibodies, in particular the compound amount (referred to as “mass dose”) contained in the tracer formulation is important for optimal uptake in tumor lesions. Large molecules, like antibodies, do not easily enter the tumor tissues due to features such as abnormal vasculature, hypoxic conditions, and increased tissue pressure, as well as due to binding sites that might need to be saturated before a biological molecule can reach the target within a tumor lesion (44–46).

**Figure 7:**
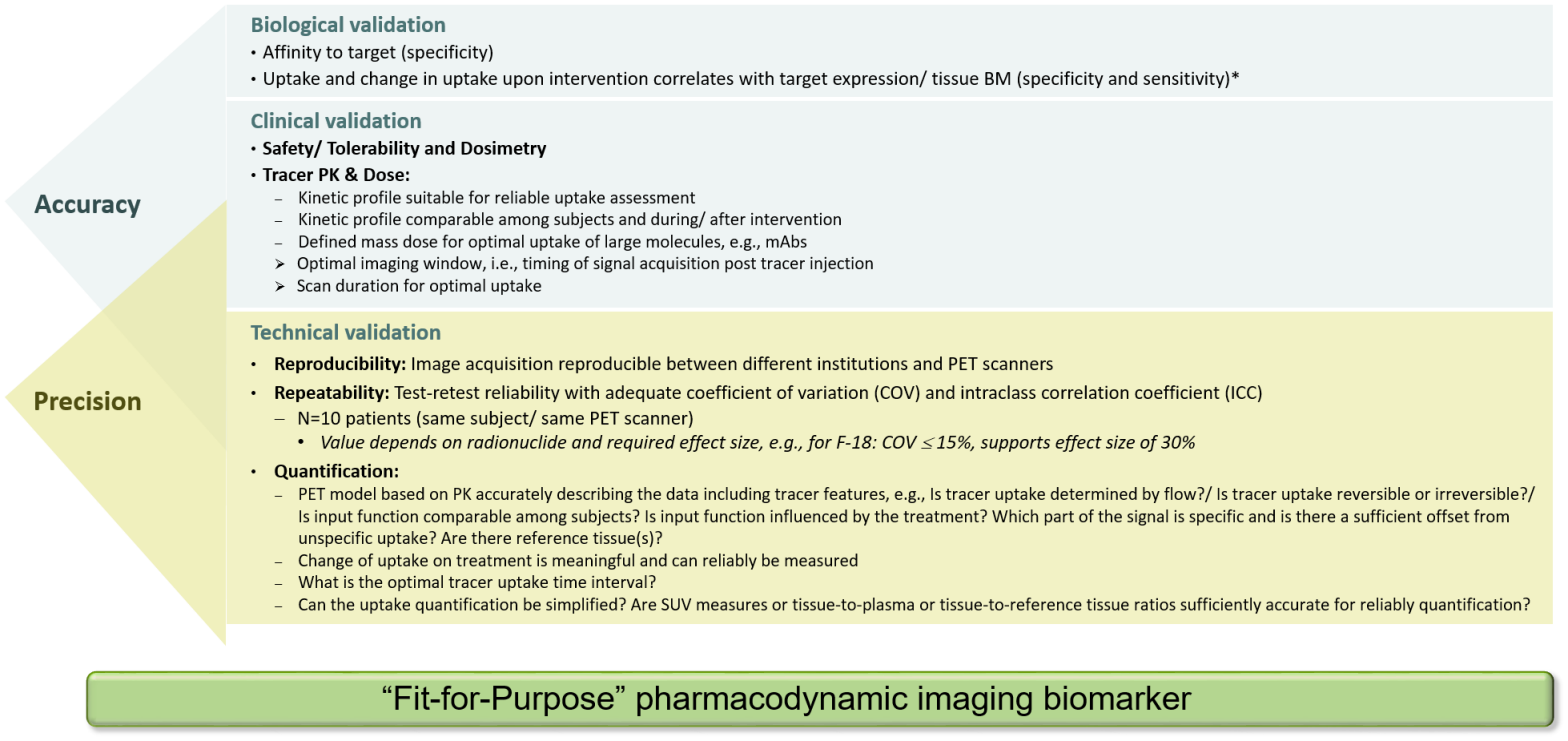
Set of data required for biological, clinical, and technical validation of a PET tracer to be considered as a ‘fit-for-purpose’ pharmacodynamic imaging biomarker for use as an exploratory biomarker in clinical development. Validation parameters to assess accuracy and precision partially overlap. *Of note, the correlation coefficient between PET ligand uptake and tissue biomarker depends on the biological behavior of the target and the percentage of sampled material containing the target.

During **technical validation** in clinical studies, a test–retest (TRT) reliability needs to be established with repeated measurements within a subject under identical conditions. For a small molecule-based PET ligand, radiolabeled with a short-lived radionuclide, the TRT reliability can be assessed by two tracer injections, each followed by a PET scan, within a short time frame, usually less than 1 week. For ^89^Zr-labeled biologics, this is not possible. Due to the long half-life, the retest cannot be performed before a minimum of 2 weeks after the first injection, a time frame where biological changes may have already occurred (47). In such cases, those evaluations might be performed in patients with long-term stable disease. To enable a ‘fit-for-purpose’ validation of the imaging BM and to assess the TRT variability, usually 10 subjects are sufficient. The accepted variability depends on the radionuclide and the uptake level in the tissue of interest. Based on available data for ^18^F-labeled tracers such as [^18^F]FDG, the accepted coefficient of variation (COV) should be in the range of approximately 15%. The higher the COV, the larger the PD effect would need to be for reliable detection in a patient. A good balance between repeatability, i.e., between- and within-subject variability, and effect size, needs to be achieved. This can be assessed using the intraclass correlation coefficient (ICC). The net PET ligand uptake at any given time is a complex interplay between delivery, uptake, retention, and clearance of the tracer (48). Thus, for a PD imaging BM, it must be demonstrated that treatment-induced changes as compared to baseline values can be measured reliably and are not influenced by possible treatment-induced changes in PET ligand PK, which can occur if metabolizing enzymes are regulated by the therapeutic drug. In Figure 7, a minimum set of recommended parameters (necessary for confirming biological, clinical, and technical validation) for a PD imaging BM to be ‘fit for purpose’ is provided. Specific aspects for validation of imaging BMs derived from radiological procedures can be found in recent publications (5, 13, 21, 49). If the imaging BM does not fulfill the requirements, additional clinical studies are indicated for a successful validation. Ideally, several aspects can be addressed in one study design. This will shorten the time frame for data generation. Importantly, upon generation of additional data through routine PET ligand development, dedicated translational studies for imaging BM validation, and the application of PET ligand in early clinical trials, the database will expand and, thus, may support additional COUs in future. An example for a clinical study design and biomarker assessment is illustrated in Figure 6. In such a study design, the uptake analysis method, thresholds for the definition of regions of interest, and test–retest reliability can be assessed and qualified. Furthermore, the study design enables to assess a correlation of the observed PET uptake with the pathology and biomarkers of the underlying tissue. In addition, addressing the complementarity between different tracers, e.g., targeting different functions of T cells, is also enabled.

### Quantification of PET tracer uptake

In certain contexts, a visual assessment of a PET scan may be sufficient; for example, when staging the disease or when assessing whether or not there is a focal uptake of the PET ligand at target-expressing sites. However, as for the human eye the perceived uptake in a region is influenced by its surroundings; the (absence of) uptake may not be unequivocally determined.

For a PD imaging BM, where changes during treatment should be demonstrated, a reliable and robust quantification metric is a prerequisite and part of the **technical validation**. To generate accurate BM data for a novel PET ligand, a thorough validation of the uptake measure considering its PK in blood or plasma and tissues needs to be performed. With a dynamic scan starting immediately after PET ligand injection, it is possible to follow its kinetics in all compartments. From modeling approaches, the various individual components that contribute to the total signal can be derived. These include specific binding, non-specific binding, and free tracer in blood or plasma and tissue compartments. To achieve this, time activity curves for tissues of interest are generated from the radioactivity concentrations measured in the relevant regions of the dynamic images. Additionally, a so-called input function from plasma or blood is determined as a measure of PET ligand supply. The data are derived from the radioactivity concentrations in the patient’s blood or plasma sampled during the scan. To exactly describe the input function for the intact tracer, the blood or plasma radioactivity data will have to be corrected for radioactive metabolites that may be formed during the scan time. Due to the slow clearance of ^89^Zr-labeled PET ligands, which are usually large biological molecules, however, a dynamic scan approach might not be feasible or informative. Instead, data describing the uptake kinetics can be generated by repeated scans during a defined time frame of days using time points matching with the kinetics of the biological molecule.

PET allows for accurate quantification of radioactivity uptake; however, the specific binding of the PET ligand to its target has to be deduced by either using compartmental modeling methods deriving delivery, transport, exchange, and binding rates in distinct tissue compartments or a reference tissue approach (48) This can be achieved by generation of ratios between uptake in target-expressing tissues and non-target containing tissues or ratios of uptake in target-expressing tissues versus blood or plasma radioactivity concentrations, as appropriate. Of note, from a single static scan, i.e., a scan of a certain duration starting at a defined time point after intravenous PET ligand injection, it is impossible to separate these components.

In oncological studies, commonly the SUV values are determined as a simplified, without taking potential differences of the PK of PET ligand into account. Importantly, as the SUV measure relates measured tissue uptake to the injected radioactivity normalized for body weight, body surface area, and lean body mass, respectively, changes in distribution volumes based on the PET ligand format are not considered (36, 38, 50). To further improve reproducibility between scanners, it is advised to calculate the SUV_peak_, instead of SUV_max_. SUV_max_ is defined as the highest SUV based on one voxel. Because of the high reproducibility between readers and an uncomplicated calculation, it is the most commonly used SUV measure. SUV_peak_ is defined as the average SUV within a 1 mL spherical volume of interest positioned to yield the highest value across all positions within the tumor. This may not necessarily be centered on the location of SUV_max_, but it is in most of the cases. SUV_peak_ is less sensitive to noise and different scanners. A clear definition of both SUV_max_ and SUV_peak_ is given in the EANM guideline for FDG PET oncology imaging and in the QIBA profile document (7, 38).

When comparing lesions between patients, it is required to take tracer supply into account. For a given injected radioactivity, the PET ligand availability depends on the clearance of the tracer from blood or plasma, including the formation of radiolabeled metabolites. Clearance may differ between patients. If this is the case, the uptake needs to be normalized to an equal tracer supply before the measured uptake can be compared. There are several examples demonstrating the importance of kinetic analyses (48, 51, 52). By use of applicable compartmental or graphical modeling approaches, such as Patlak linearization, as typically applied for small molecules (53) or by the generation of tumor-to-plasma ratios or net rates of irreversible uptake applied for large molecules like antibodies (54, 55), the PK of the PET ligand is integrated allowing a reliable quantification. Another important aspect is the knowledge on target binding properties, i.e., whether the tracer compounds bind reversible or irreversible to its target and whether the internalized radionuclide residualizes in cells (such as ^89^Zr) or not (such as ^124^I) (56, 57). Once thorough uptake quantification analysis has been performed and robust data have been generated, simplified approaches that will not compromise the accuracy of the measurement can be tested against the developed models. There is literature from neurological applications using small molecule-based PET ligands that points to the importance of an uptake metric that is related to target binding and is not influenced by the presence of other, target-unrelated tracer uptake in the region of interest (48).

**Figure 8:**
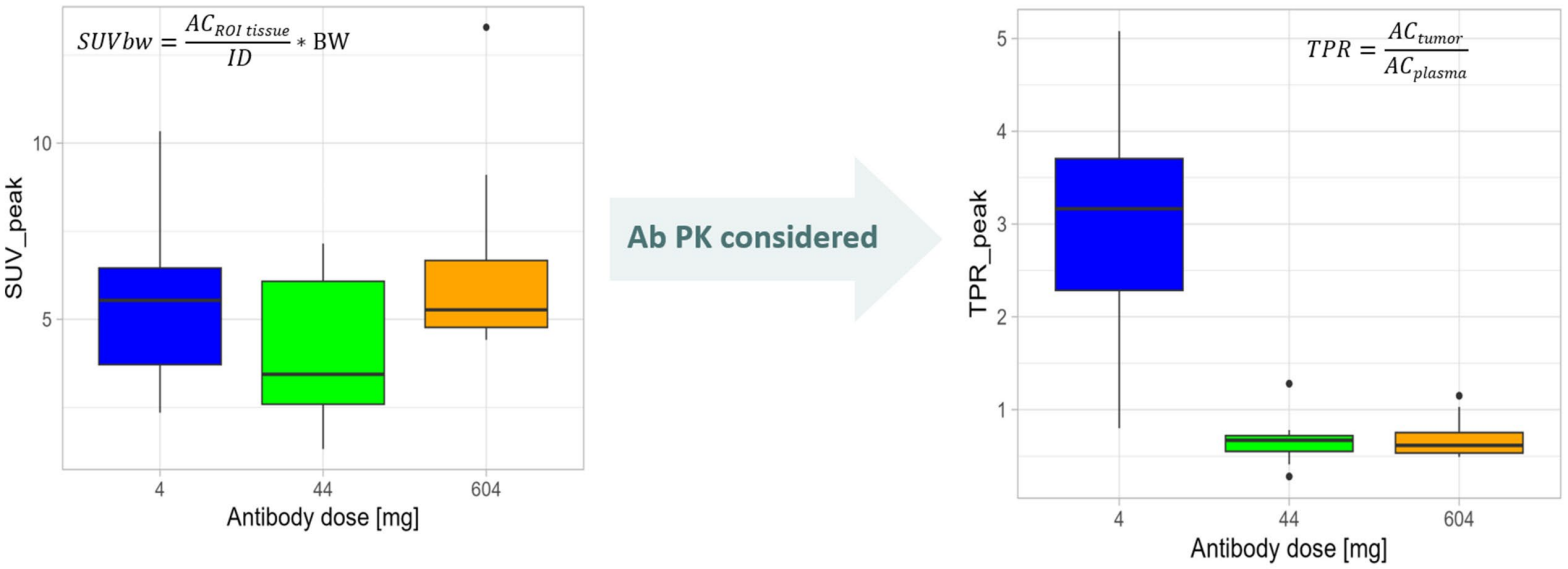
Need to quantify tracer uptake considering PET tracer kinetics demonstrated using an mAb radiolabeled with ^89^Zr as an example. The change in mAb dose is not reflected by SUV_peak_ values that measure the signal in a 1-mm sphere around the highest SUV value pixel, i.e., the region of interest (ROI), in a semiquantitative manner by relating the uptake in the region of interest (radioactivity concentration (AC) in ROI_tissue_) to the injected dose (ID) and normalized to, e.g., body weight (BW; see formula) not regarding the tracer supply in blood and plasma. When taking the Ab PK into consideration. i.e., assessing tumor-to-plasma ratios (TPR) by normalizing the activity concentration in the tumor ROI (AC_tumor_) for the plasma activity concentration (AC_plasma_; see formula), differences in tumor uptake between antibody doses were demonstrated. Plots are generated based on data from Miedema et al. (43).

In order to assess TME features, often PET ligands based on large molecules have been utilized, having different PK profiles compared to small molecules. The importance of using kinetically informed uptake metrics was impressively demonstrated for monoclonal antibodies (mAbs) (43, 58). Three main PK properties need to be considered when deciding on a reliable quantification metrics for target-mediated mAbs uptake: (1) mAbs extravasate rather slowly into tissue interstitial spaces, followed by faster clearance via lymphatic drainage of the tissue. This leads to an overall volume of distribution at steady state in which the tracer can reside in the range of 6 to 15 L, at a central compartment volume of approximately 3 L, similar to the plasma (59); (2) due to the potential presence of an antigen sink or due to target mediated drug disposition, plasma PK may be mass dose-dependent and (3) mAb elimination occurs mostly through intracellular catabolism by lysosomal degradation (60). Thus, as ^89^Zr resides in the lysosomes of the cells, the elimination of large biological molecules, such as mAbs, leads to irreversible ^89^Zr accumulation through target-independent (non-specific) catabolic processes. On the other hand, target-dependent irreversible ^89^Zr accumulation occurs through receptor–mAb interactions (specific). For the target non-specific uptake process, the baseline uptake of ^89^Zr-labeled mAbs in a selection of normal tissues was calculated from an experimental assessment (61, 62). The impact for quantification metrics acknowledging the PK properties of the PET ligand has been demonstrated in a study using an ^89^Zr-labeled LAG-3 antibody as a PET ligand administered together with three different mAb doses. When applying SUVs for uptake assessment no difference in uptake between the dose cohorts was found (43). However, when assessing uptake by tissue over plasma ratios or the net rate of irreversible uptake using a well-known PET modeling approach, i.e., Patlak transformation as a graphical analysis method, clear differences in uptake can be detected (53, 63) (Figure 8).

### Characterization of the TME using MRI

Pathophysiological features of the TME can also be assessed by MRI, which offers several clinically useful biomarkers, notably oxygen-enhanced MRI biomarkers of hypoxia (64), dynamic contrast-enhanced MRI biomarkers of perfusion and permeability (23), and amide proton transfer (a proxy for protein concentration) (65). While these biomarkers are not specific for immune cell components of the TME, they provide important context, as hypoxia and inadequate perfusion can affect efficacy in immuno-oncological treatments. In addition, MRI offers other investigational biomarkers, for example, phagocytosed iron oxide nanoparticles to assess cell trafficking (66) and lymph node involvement (67).

MR biomarkers based on diffusion-weighted imaging (DWI) might be very useful in the early development of immune-oncological drugs (68, 69). DWI can be acquired on most clinical MRI scanners (including PET-MRI scanners) and does not involve the use of contrast agents or other special equipment. DWI reflects the degree of diffusion of water molecules through different tissues. Because diffusion is impeded within cells, and across cell membranes, DWI is interpreted as a biomarker of cellularity. The biomarker typically derived from such a scan reported is the apparent diffusion coefficient (ADC). Effective anti-cancer therapies typically decrease the amount of tumor cells, enhance diffusion, and increase ADC, a treatment-agnostic response but common in immune-oncology (70, 71). It has been suggested that effective immunotherapy with acute immune cell infiltration into the tumor will induce a paradoxical acute reduction in ADC (72, 73), analogous to the increase in tumor lesion glucose consumption by infiltrating immune cells sometimes seen in [^18^F]FDG PET (74) or pseudoprogression due to the same event observed in MRI or CT (75, 76). Indeed, this phenomenon has been reported in a recent study assessing both CD8^+^ cell infiltration and ADC by PET-MRI imaging, showing an increase in immune cell infiltration while the ADC decreases at the same time (16). More studies are lacking, perhaps partly because of the difficulty in timing the acquisition to coincide with increased cellularity from immune cell infiltration but before massive cell death. ADC is widely used as a PD imaging BM, especially in single-center studies. For multicenter trials, harmonization is essential to standardize data from different set-ups and models of scanners with different gradient performances and pulse sequences (68). To assure the reliability of the ADC data, a double-baseline DWI assessment before treatment starts could be introduced to determine the intra-patient variability.

Solid tumors often exhibit regional hypoxia, which is immunosuppressive (77). Imaging biomarkers that assess tumor hypoxia might therefore be useful as predictive biomarkers for patient selection or stratification. They could also be useful as response biomarkers to assess the efficacy of combination therapies that aim to alleviate hypoxia to improve immunotherapy. In this setting, the most useful MR biomarkers require the administration of a paramagnetic contrast agent, i.e., a substance, which modulates the nuclear magnetic relaxation properties of the tumor tissue. The tumor is imaged, usually dynamically over a few minutes before and during the administration of the contrast agent. Contrast agents include gadolinium chelates used routinely in radiology (gadoterate, gadobutrol, gadoteridol, and newly introduced gadopiclenol).

For gadolinium-based contrast agents, T_1_-weighted images are acquired sequentially every few seconds and analyzed voxelwise with a compartmental model (78). The commonly used Tofts model yields a transfer constant K^trans^/s^−1^, which reflects blood flow and endothelial permeability and, thus, informs on tumor perfusion properties. Alternatively, the fraction of enhancing voxels can be used to derive a vascularised tumor volume/ml. As a pharmacodynamic biomarker, K^trans^ can be regarded as ‘fit-for-purpose’ in early-phase drug development as it has been extensively and successfully used particularly in trials of anti-angiogenic drugs (23). Of note, dynamic contrast-enhanced MRI biomarkers of perfusion and permeability have been associated with progression vs. pseudoprogession in multiple studies involving immune and other therapies (79).

A more direct assessment of tumor hypoxia can be obtained from oxygen-enhanced MRI, which does not require a contrast agent (64, 80). Dissolved oxygen is paramagnetic, whereas oxygen bound to heme is not. In tumor regions that are already adequately oxygenated and haems are nearly saturated, hyperoxia leads to an increase in dissolved paramagnetic oxygen, which can be detected in T_1_-weighted MRI, just as for gadolinium. However, in hypoxic regions, where haems are mostly unsaturated, hyperoxia simply reduces the unsaturation, causing a negligible increase in dissolved oxygen and no change in T_1_-weighted MRI. These regions, which are refractory to hypoxia, correspond to pathologically hypoxic regions. More specificity can be gained by distinguishing perfused hypoxia refractory (where perfusion is assessed using gadolinium-based dynamic contrast-enhanced MRI), from non-perfused hypoxia refractory, the latter often corresponding to regions of necrosis (81). Oxygen-enhanced biomarkers have been shown to respond to therapeutic intervention in animal models and in lung cancer patients (82).

So far, there are few studies of dynamic contrast-enhanced MRI and oxygen-enhanced MRI-based biomarkers in immuno-oncology. However, in the pharmacodynamic COU, these biomarkers are generally ‘fit-for-purpose’. They employ regulatory-approved contrast agents, with good literature evidence for imaging-pathology correlation and effect sizes. Repeatability should be verified where possible using double-baseline scans, which are usually ethically acceptable because of the absence of ionizing radiation. Acquisition and analysis should be standardized, particularly in multicenter trials involving different makes and models of scanners. Several PET tracers to assess tumor hypoxia are currently being investigated in both preclinical and clinical studies. However, so far no optimal tracer to assess cellular hypoxia has been established (83).

### Importance of imaging system harmonization and central assessments

For high data quality and reliability, reproducible and accurate quantitative readouts on the PD imaging BMs, a standardized and harmonized image acquisition, high image quality, and an aligned analysis procedure are essential. To achieve this, all clinical imaging procedures such as patient preparation, tracer specifications, image processing, and analysis need to be harmonized between sites. For a multicenter setting, these processes are ideally supported by a central imaging vendor. This will reduce inter- and intra-institutional variability.

It is important to harmonize and standardize the scanning procedures, PET systems, MRI sequences, and data from different models and makes of scanners with different gradient performance and pulse sequences, as applicable for the trial. There are several guidelines available, such as the *Quantitative Imaging Biomarker Alliance of the Radiological Society of North America* (QIBA) (38) and the *European Association of Nuclear Medicine* (EANM) *Research Ltd*. (EARL) (84). The EARL accreditation program is an example for PET system harmonization. The scanners will be accredited for ^18^F and additional radionuclides, such as ^68^Ga and ^89^Zr, through a central authorized expert body. This assures comparable scanner performance through the harmonization of reconstruction procedures across multiple sites with different scanner models. Detailed information can be found on the EARL website. In addition, and not implemented so far into the accreditation procedure, well counters need to be calibrated in case blood and plasma samples need to be measured to get calibration factors to assure comparability of blood and plasma radioactivity data. This is important if a blood or plasma input function is derived or for tissue-to-blood/plasma ratios as read-out parameters for the intended PD imaging BM.

## Outlook

To ensure a seamless implementation of imaging BMs into clinical trials and potentially further into clinical routine, developers of the PET ligands, manufacturers of imaging devices, pharmaceutical companies, and academic centers performing such studies should work together. In this respect, pre-competitive consortia are supportive. For example, *Immune-Image* within the *Innovative Medicines Initiative* (IMI) (https://www.imi.europa.eu/projects-results/project-factsheets/immune-image) in Europe has the objective to develop novel, non-invasive imaging strategies for assessing immune cell activation and dynamics in oncology and inflammatory disease, in animal models and patients. Another precompetitive consortium is the *FNIH Biomarkers Consortium* (FNIH BC) bringing partners from pharmaceutical companies and academia together with the aim to identify, develop, and qualify potential BMs to improve drug development and regulatory decision-making. Within the FNIH BC, an *Immune Response Imaging Working Group* has been established pursuing pre-competitive trials to support additional characterization of PET ligands.

With many new immune therapies for cancer treatment being developed, the availability of PD imaging BMs in addition to traditionally applied tissue and blood-based BMs will provide a deeper insight into whether the mode of action of expected drug translates into effects observed in the patients. PD imaging BMs enable the assessment of intra- and inter-tumoral heterogeneity as well as whole-body changes in PET tracer uptake. This has the potential to answer questions about applications of treatment combinations. With such imaging BMs qualified for a specific COU, decisions in the drug development process can be facilitated, strongly supporting an efficient drug development.

## Author contributions

JE: Conceptualization, Project administration, Writing – original draft, Writing – review & editing, Investigation. IB: Conceptualization, Investigation, Visualization, Writing – review & editing. JW: Investigation, Methodology, Writing – review & editing. MH: Methodology, Writing – review & editing. RB: Methodology, Resources, Writing – review & editing. AW: Writing – review & editing. AT: Conceptualization, Investigation, Methodology, Project administration, Resources, Writing – original draft, Writing – review & editing. CM: Conceptualization, Resources, Writing – original draft, Writing – review & editing.
